# Relaxor Ferroelectricity Enables Enhanced Thermoelectric Performance in In_2_Se_3_‐Alloyed GeTe

**DOI:** 10.1002/advs.76578

**Published:** 2026-07-20

**Authors:** Yuting Zhang, Yang Li, Jianguo Chen, Kai Guo, Jun Luo, Jiye Zhang

**Affiliations:** ^1^ School of Materials Science and Engineering Shanghai University Shanghai China; ^2^ School of Physics and Materials Science Guangzhou University Guangzhou China; ^3^ Guangdong Provincial Engineering Research Center for Materials under Extreme Service Environments Guangzhou University Guangzhou China; ^4^ Interdisciplinary Materials Research Center School of Materials Science and Engineering Tongji University Shanghai China

**Keywords:** ferroelectricity instability, GeTe, high average *zT* value, thermoelectric materials

## Abstract

The intuitive challenge in combining ferroelectric and thermoelectric materials lies in their diametrically opposed electrical conductivity requirements. As a narrow‐band‐gap semiconductor with spontaneous ferroelectric distortion, GeTe serves as an ideal paradigm for investigating the interplay between ferroelectricity and thermoelectricity, yielding novel strategies for synergistically optimizing the electrical and thermal transport properties of thermoelectric materials. In this work, a non‐equimolar (2:3) In^3+^/Se^2−^ alloying strategy was implemented to deliberately introduce Ge vacancies in GeTe under an electrical neutrality condition. As a stronger perturbation to both lattice periodicity and local charge neutrality, Ge vacancies can effectively induce relaxor ferroelectricity in GeTe and thus broaden the temperature window of its minimum thermal conductivity. Combining the unique role of Ge vacancies in stabilizing the cubic phase of GeTe, a broadened temperature range of optimized electrical transport performance was simultaneously realized. Ultimately, a trade‐off between electrical and thermal transport properties in an extended temperature range was achieved in (GeTe)_1‐2_ *_x_* (In_2_Se_3_)*_x_* (*x* = 0.02–0.04) samples, yielding an optimized average *zT* value exceeding 1.0 within 330–625 K.

## Graphical abstract

A non‐equimolar In^3+^/Se^2−^ alloying strategy creates Ge vacancies in GeTe, inducing relaxor ferroelectricity that broadens the low thermal conductivity window. Simultaneously, cubic phase stabilization broadens optimal electrical transport, achieving an average *zT* > 1.0 over 330–625 K.

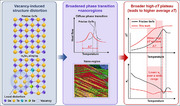

## 1 Introduction

In recent decades, the growing energy demand has led to accelerated progress in renewable energy technology. Thermoelectric (TE) materials, which can directly convert heat and electricity based on the Seebeck and Peltier effects through charge carrier transport, play an important role in waste heat recovery and solid‐state refrigeration [1, 2]. A dimensionless figure of merit *zT* can represent the performance of TE materials at a specific temperature, $zT\;=\frac{S^{2}\sigma T}{\kappa_{\mathrm{tot}}}$, where *S*, *σ*, *T*, *κ*_tot_ are the Seebeck coefficient, electrical conductivity, absolute temperature, and total thermal conductivity *κ*_tot_ (*κ*_tot_ = *κ*_e_ + *κ*_L_) [3], where *κ*_e_ and *κ*_L_ represent the electronic and lattice components of thermal conductivity, respectively [4]. The performance of a practical thermoelectric device, however, is not dictated by a single *zT* value at a fixed temperature. Under real operating conditions, a significant temperature gradient (Δ*T*) exists across the material. Given the strong temperature dependence of the transport properties (*S*, *σ*, *κ*), the local *zT* varies considerably throughout the device. Therefore, the overall conversion efficiency (*η*) is determined by the average *zT* (*zT*_avg_) over the entire operational temperature range, rather than by the peak *zT* (*zT*_max_) alone [5]. Practically, optimizing *zT* is often challenging because the key transport parameters mentioned above are strongly coupled and mutually constraining. Therefore, improving *zT*_avg_ of a TE material hinges on achieving an ideal compromise between electrical transport performance and the relatively independent *κ*_L_ across a broad temperature range.

Currently, research on TE materials focuses on exploring new material systems and optimizing existing ones, spawning high‐performance thermoelectric materials such as SnSe [6], Cu_2_Se [7], SnS [8], Mg_3_(Sb,Bi)_2_ [9] and GeTe [10, 11, 12], and developing manipulation strategies like band engineering for optimizing electrical transport performance and phonon engineering for minimizing *κ*_L_ [13, 14]. Among them, traditional phonon engineering strategies primarily focus on introducing multi‐scale phonon scattering centers, such as point defects, dislocations, nanoprecipitates, and grain boundaries [15, 16, 17]. However, these scattering mechanisms often concurrently impede charge carrier mobility, leading to a detrimental trade‐off between electrical and thermal transport properties [18]. Therefore, introducing novel mechanisms that can cooperatively modulate electrical‐thermal transport, rather than independently optimizing one at the expense of the other, is imperative. Ferroelectric materials offer a promising avenue in this regard, in which the appearance of ferroelectric order is characterized by the softening of polar transverse optical phonons, bringing their energy comparable to that of heat‐carrying acoustic phonons [19, 20]. This dramatically intensifies the scattering of acoustic phonons via the inherently strong acoustic‐optical coupling, leading to a suppression of *κ*_L_ that manifests as a distinct minimum in thermal conductivity at the ferroelectric transition temperature [21].

However, the fundamental challenge in utilizing such an effect stems from the contradictory demands for electrical conductivity in traditional ferroelectric versus thermoelectric materials [22, 23]. This historical divergence has long prevented the beneficial interplay between ferroelectric and thermoelectric properties. Remarkably, GeTe may bridge the gap between these two seemingly incompatible functionalities. As a narrow‐bandgap ferroelectric semiconductor, it inherently combines the electrical conductivity required for thermoelectric performance with the strong anharmonicity and structural instability of typical ferroelectrics [24]. Before transforming into the cubic rock‐salt structure (*c*‐GeTe, space group: $Fm\bar{3}m$) at temperatures exceeding 700 K, GeTe possesses a rhombohedral phase (*r*‐GeTe, space group: *R*3*m*), which can be viewed as a slight tensile distortion of the cubic phase along the body diagonal [111] direction [25, 26]. Such distortion, induced by the stereochemical activity of the Ge^2+^ lone‐pair electrons, also drives an off‐center displacement of Ge, thereby breaking the inversion symmetry of the crystal and leading to the formation of a ferroelectric phase and polarization domains [27]. Furthermore, the degree of rhombohedral distortion, characterized by the rhombohedral angle (deviating from 90° to approximately 88.2°), directly dictates the degeneracy of the electronic bands at the L and Σ points [25].

Recently, Biswas et al. demonstrated that in the BiSe and Pb co‐doped GeTe system, the inhomogeneous ferroelectric instability, enabled by spatially localized polar distortions induced near the dopant sites, can broaden the region of minimum thermal conductivity (*κ*_min_), achieving glassy thermal transport over a wide temperature range [28]. Crucially, while such local instability can effectively disrupt the phonon propagation, it maintains a largely intact conductive electronic framework. Therefore, engineering local ferroelectric instability may provide a powerful approach for optimizing the thermoelectric performance of GeTe over a wide temperature range.

**FIGURE 1:**
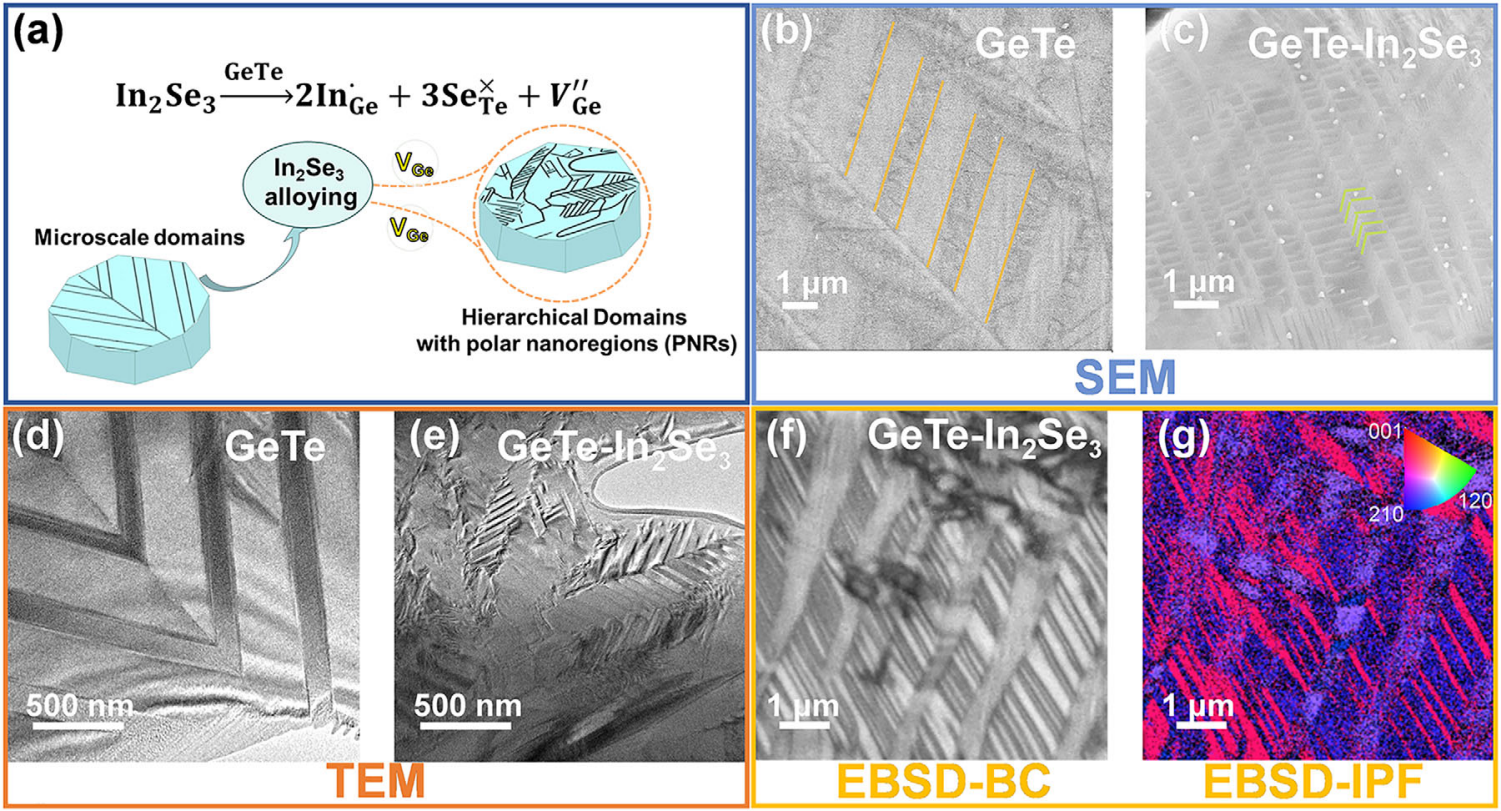
(a) Schematic illustration of the domain structure evolution of In_2_Se_3_ alloyed GeTe. (b,c) Backscattered electron (BSE) images of GeTe and (GeTe)_0.96_(In_2_Se_3_)_0.02_, respectively. (d,e) Bright‐field TEM images of GeTe and (GeTe)_0.96_(In_2_Se_3_)_0.02_, respectively. (f,g) Electron backscatter diffraction band contrast (EBSD‐BC) and electron backscatter diffraction inverse pole figure (EBSD‐IPF) maps of (GeTe)_0.96_(In_2_Se_3_)_0.02_.

Herein, we implemented a non‐equimolar (2:3) In^3+^/Se^2−^ alloying strategy to manipulate Ge vacancies in GeTe (schematically illustrated in Figure 1a). Due to the 1:1 ratio of cations to anions and the requirement for charge neutrality in GeTe, substituting three Ge^2+^ ions with two In^3+^ ions introduces one Ge vacancy. As a stronger source of perturbation in both lattice periodicity and local charge neutrality, the introduction of Ge vacancies induces relaxor ferroelectric behavior in GeTe, including polar nanoregions (PNRs) and diffuse phase transition, thereby extending the temperature range of its *κ*_min_. On the other hand, the increasing concentration of Ge vacancies subtly drives the structure of GeTe toward higher symmetry (manifested by the increased rhombohedral angle from 88.2° to 89.5°) and thus enhances the band degeneracy. Furthermore, it is generally recognized that a deliberate increase of lattice vacancies at Ge‐sites promotes the dissolution of Ge precipitates [29]. Combined with the increase in the electronegativity of the anionic lattice due to Se substituting Te, the hole concentration of GeTe was substantially reduced [30]. Consequently, a synergistic optimization of the power factor and the lattice thermal conductivity in an extended temperature range was achieved in (GeTe)_1‐2_ *_x_* (In_2_Se_3_)*_x_* (*x* = 0.02–0.04) samples, yielding an optimized average *zT* value exceeding 1.0 within the temperature range of 330–625 K.

## 2 Experimental Section

### 2.1 Raw Materials and Sample Preparation

High‐purity Ge (99.999%), Te (99.999%), In (99.999%), and Se (99.999%) were weighed according to the nominal compositions (GeTe)_1‐2_ *_x_* (In_2_Se_3_)*_x_* (*x* = 0, 0.01, 0.02, 0.03, 0.04, 0.05, 0.06, 0.07), which were then sealed in evacuated quartz ampoules. The mixtures were slowly heated to 673 K and held for 4 h, followed by heating to 1273 K and dwelling for 24 h. Subsequently, the melts were cooled to 973 K at a rate of 2 K min^−1^ and rapidly quenched in cold water. The obtained ingots were annealed at 873 K for 72 h to ensure compositional homogeneity, and were ground into fine powders. The powders were loaded into graphite dies with a diameter of 10 mm and hot pressed at 873 K under 55 MPa for 30 min. All sintered bulk samples exhibited relative densities above 95%. The sintered disks were used for thermal transport measurements. Dense bar‐shaped samples with dimensions of 3 × 3 × 8 mm were cut from the disks for electrical transport measurements.

### 2.2 Structural and Transport Property Characterizations

Phase identification and crystal structure analysis were carried out by powder x‐ray diffraction (XRD, Rigaku, Japan) using Cu Kα radiation (*λ* = 1.541854 Å) at 40 kV and 30 mA. Rietveld refinement was performed using the GSAS‐II software package [31] to determine the interaxial angle and lattice parameters. The electrical resistivity and Seebeck coefficient were simultaneously measured using a ZEM‐3 integrated electrical transport measurement system (ULVAC‐RIKO, Japan) from 300 to 773 K. Thermal diffusivity (*D*) was collected by the laser flash method using an LFA 457 instrument (Netzsch, Germany). The specific heat capacity *C*_p_ was estimated from the Dulong‐Petit law. The total thermal conductivity (*κ*_tot_) was calculated according to *κ*_tot_ = *ρC*p*D*, where *ρ* is the sample density determined by the Archimedes method. The morphology and microstructure of the bulk samples were characterized by field‐emission scanning electron microscopy (FESEM, Gemini 300, Zeiss, Germany) equipped with an energy‐dispersive x‐ray spectrometer (EDS, X‐Max 51, Oxford Instruments, UK) and an electron backscatter diffraction detector (EBSD, Aztec Nordlys Max3). The evolution of ferroelectric domain structure in the submicron dimension is further observed under a scanning transmission electron microscopy (STEM, JEM‐F200, JEOL, Japan). The room‐temperature Hall coefficient (*R*_H_), carrier concentration (*n*_H_), and Hall mobility (*µ*_H_) were measured using a physical property measurement system (PPMS, Quantum Design, USA) under a reversible magnetic field of 3 T.

## 3 Results and Discussion

**FIGURE 2:**
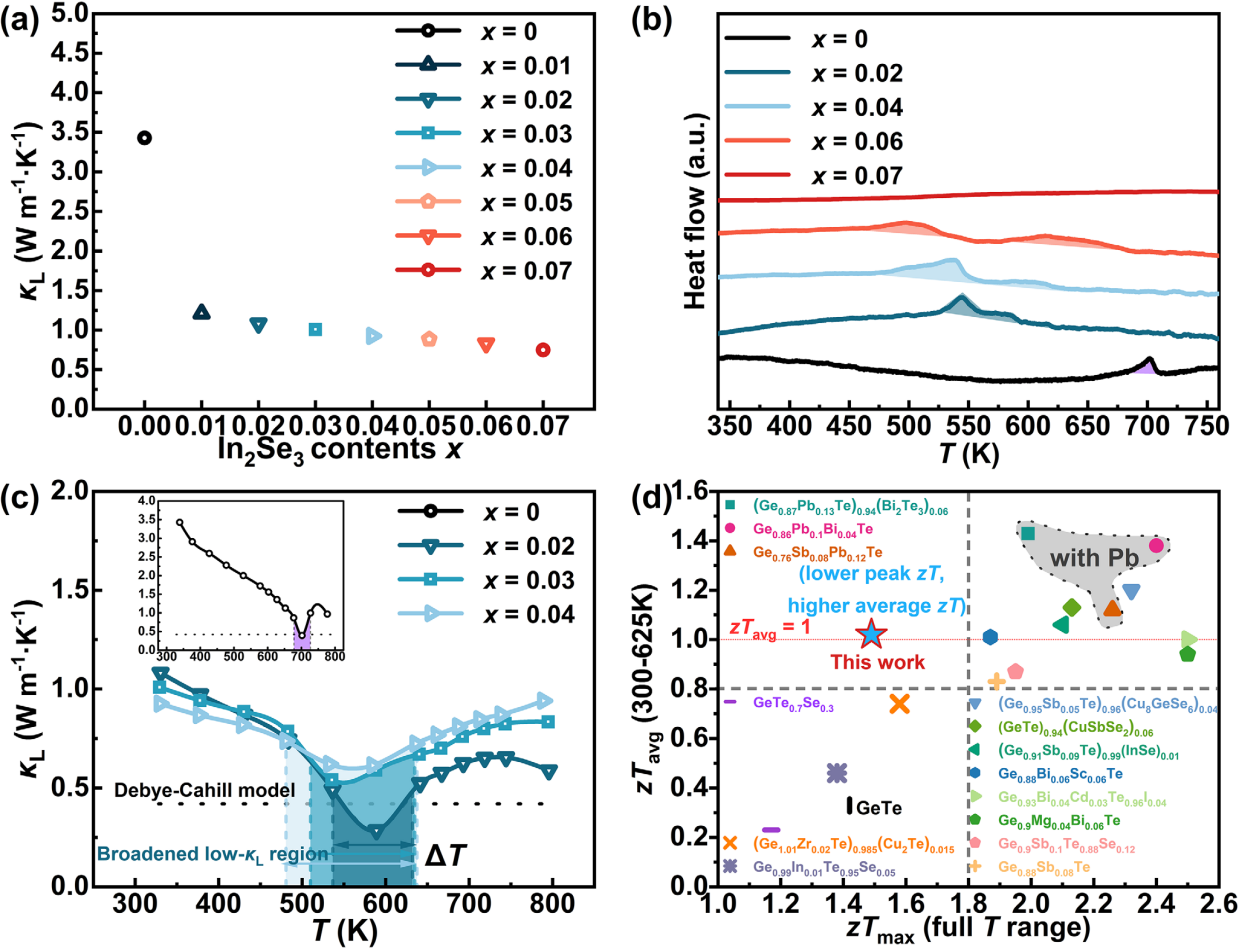
(a) Room‐temperature lattice thermal conductivity (*κ*_L_) as a function of In_2_Se_3_ content *x* in (GeTe)_1‐2_ *_x_* (In_2_Se_3_)*_x_* (*x* = 0–0.07). (b) Differential scanning calorimetry curves of (GeTe)_1‐2_ *_x_* (In_2_Se_3_)*_x_* (*x* = 0, 0.02, 0.04, 0.06 and 0.07). (c) Temperature dependence of lattice thermal conductivity (*κ*_L_) for (GeTe)_1‐2_ *_x_* (In_2_Se_3_)*_x_* (*x* = 0, 0.02, 0.03, 0.04). (d) Comparison of the average *zT* (*zT*_avg_) values in the temperature range of 330 ∼ 625 K and the maximum *zT* over the full measured temperature range between this work and previously reported literature [29, 30, 33, 34, 35, 36, 37, 38, 39, 40, 41, 42, 43].

Figure 1a schematically illustrates the domain structure evolution of the GeTe matrix upon In_2_Se_3_ alloying, highlighting the enhanced ferroelectric domain refinement and structural inhomogeneity, as corroborated by multiple microstructural characterization techniques. Under backscattered electron (BSE) imaging in SEM (Figure 1b,c), GeTe clearly displays large, well‐ordered ferroelectric domains, which become fragmented and irregular after the addition of In_2_Se_3_. To more clearly reveal the evolution of the domain structure, the microstructures of the samples were further examined by transmission electron microscopy (TEM). As shown in Figure 1d,e, distinct microstructural differences are observed between pristine GeTe and (GeTe)_0.96_(In_2_Se_3_)_0.02_. At the same magnification, the well‐defined, regularly arranged herringbone‐like ferroelectric domains characteristic of GeTe undergo a dramatic reduction in size upon introduction of a small amount of In_2_Se_3_, evolving into a hierarchical structure comprising nanoscale polar regions within the alloyed sample. Concurrently, abundant dislocations are also observed in the TEM images. As further shown in Figure S1, higher‐magnification TEM images reveal refined nanoscale domain features and local structural inhomogeneity in the In_2_Se_3_‐alloyed GeTe sample. The electron backscatter diffraction (EBSD) results of the (GeTe)_0.96_(In_2_Se_3_)_0.02_ sample shown in Figure 1f (band contrast image, EBSD‐BC) and Figure 1g (inverse pole figure, EBSD‐IPF) further reveal that submicron‐scale stripe‐like structures are present, indicating significant refinement of the domain structures. Additionally, the ferroelectric domain orientations become randomized, suggesting enhanced local orientation perturbations. A wider‐field EBSD map in Figure S2 further shows that these refined domain structures and local orientation perturbations are distributed over a broader region rather than being merely local features. Ferroelectric domain refinement, orientational randomization, and the introduction of dislocations collectively suppress phonon transport, leading to a pronounced reduction in the average sound velocities (*v*_a_, Figure S3) and room‐temperature *κ*_L_ of the In_2_Se_3_‑alloyed sample compared to that of pristine GeTe (Figure 2a). Furthermore, the emergence of PNRs in the In_2_Se_3_‐alloyed sample is reminiscent of the domain structure of relaxor ferroelectrics [32], which is also manifested in the diffuse nature of the ferroelectric phase transition.

As shown in Figure 2b, the endothermic peaks in the differential scanning calorimetry (DSC) curves gradually evolve from sharp to multimodal, broad, and flattened with increasing In_2_Se_3_ content, signaling a progressively diffuse phase transition that extends over a progressively wider temperature range. Furthermore, the onset of the endothermic peak in the DSC curves progressively shifts toward room temperature with increasing In_2_Se_3_ contents, until it completely disappears at *x* = 0.07. For the *x* = 0.07 sample, no obvious endothermic peak can be distinguished in the DSC curve. This result does not necessarily indicate the complete disappearance of the ferroelectric phase transition. Instead, it suggests that the transition is strongly weakened and becomes highly diffuse, making the corresponding thermal signal difficult to resolve by DSC. This indicates that the incorporation of In_2_Se_3_ may increase the interaxial angle of GeTe, thereby promoting its lattice symmetry [44]. Therefore, it is anticipated that the In_2_Se_3_‐alloyed sample will exhibit a low thermal conductivity regime over a broader temperature range than pristine GeTe. Actually, it can be seen in the inset of Figure 2c, the temperature dependence of *κ*_L_ for pristine GeTe exhibits a very narrow dip, which corresponds exactly to the sharp endothermic peak at 700 K in the corresponding DSC curve. With the incorporation of In_2_Se_3_, the valleys in *κ*_L_ curves for samples with ferroelectric distortion (*x* = 0.02, 0.03 and 0.04) progressively broadens (Figure 2c and Figure S4), giving rise to a broad low‐thermal‐conductivity region in terms of temperature. The relaxor behavior of In_2_Se_3_‐alloyed GeTe samples can be ascribed to the Ge vacancies created by the non‐equimolar (2:3) In^3+^/Se^2−^ substitution. Here, In_2_Se_3_ alloying triggers a charge‐neutral defect reaction in which two In atoms replace two Ge atoms, three Se atoms replace three Te atoms, and one Ge vacancy is generated. The defect reaction equation can be expressed as:


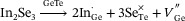




In fact, extensive research on relaxor ferroelectrics has demonstrated that, compared with substitutional doping, the introduction of cation vacancies not only generates chemical pressure and lattice distortion but also introduces additional perturbations to the long‐range Coulomb potential and stronger charge pinning effects [45, 46, 47]. Thus, as a stronger source of perturbation, cation vacancies are more effective in promoting structural inhomogeneities and the formation of PNRs [35]. More importantly, the introduction of abundant Ge vacancies through isovalent solid solution with In_2_Se_3_ may potentially drive the transformation from *r*‐GeTe to *c*‐GeTe with higher symmetry (discussed below), which may increase the band degeneracy and thus optimize the electrical transport performance. Consequently, a trade‐off between the electrical and thermal transport properties can be accomplished across a broad temperature regime in (GeTe)_1‐2_ *_x_* (In_2_Se_3_)*_x_* (*x* = 0.02–0.04) samples.

The *zT*_avg_ values of all compared samples were calculated over the same temperature range of 330–625 K, where the In_2_Se_3_‐alloyed GeTe samples show suppressed *κ*_L_ and favorable power factors, enabling a fair comparison of average thermoelectric performance. As illustrated in Figure 2d, although the *zT*_max_ (∼1.6) of the samples in this work is lower than that of some previously reported high‐performance GeTe‐based systems, the *zT*_avg_ still exceeds 1.0 without Pb incorporation, demonstrating competitive thermoelectric performance in terms of *zT*_avg_ among Pb‐free GeTe materials. Notably, the *zT*_avg_ is also significantly higher than that of the solely In‐doped or solely Se‐doped samples, suggesting the beneficial role of Ge vacancies in the synergistic optimization of thermoelectric performance.

**FIGURE 3:**
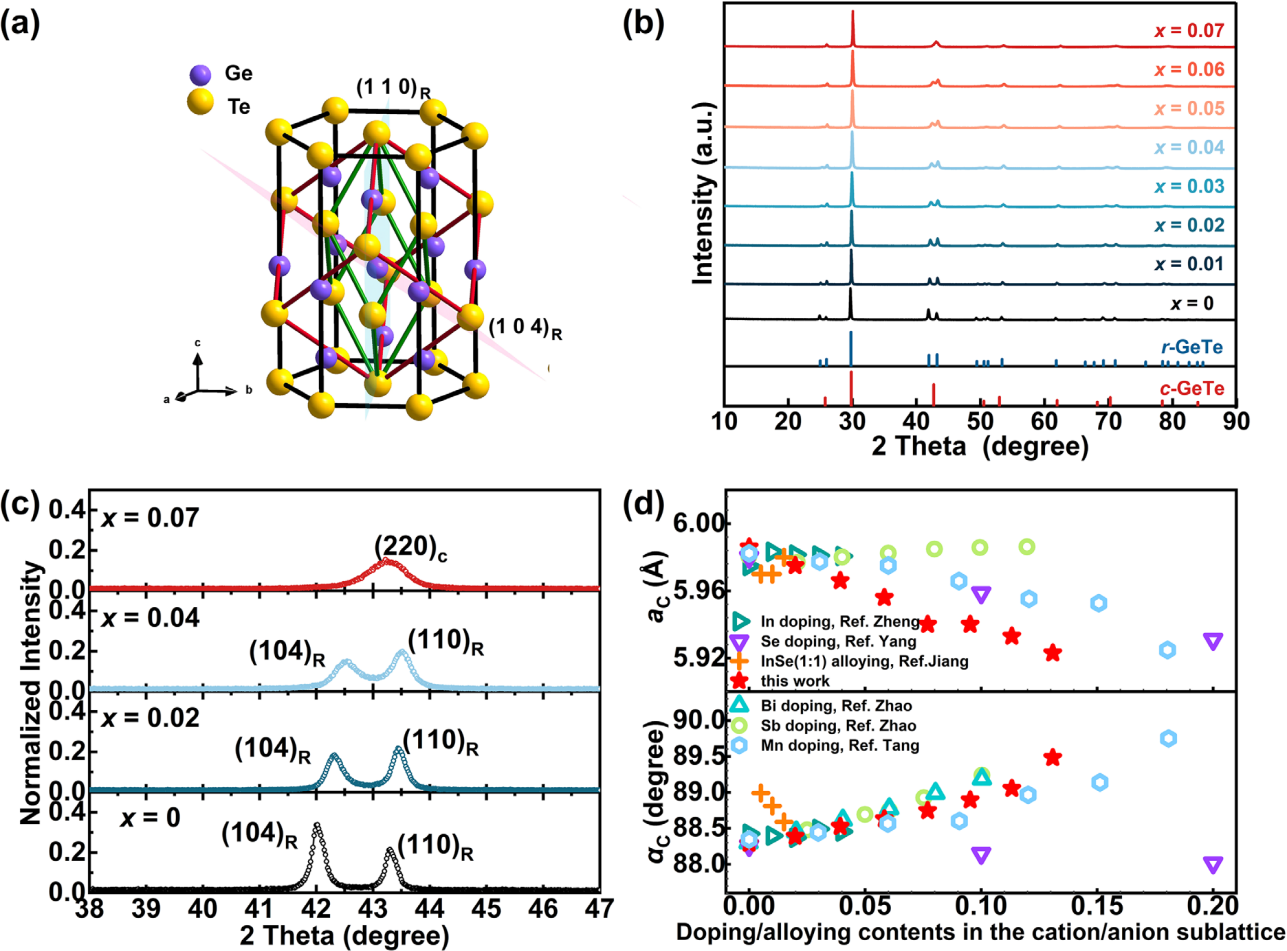
(a) Schematic crystal structures of GeTe at room temperature represented in the hexagonal lattice (black), pseudo‐cubic lattice (red), and rhombohedral lattice (green). (b) Room‐temperature powder XRD patterns of (GeTe)_1‐2_ *_x_* (In_2_Se_3_)*_x_* (*x* = 0, 0.01, 0.02, 0.03, 0.04, 0.05, 0.06, 0.07). (c) Evolution of diffraction peaks around 2*θ* ≈ 43° for (GeTe)_1‐2_ *_x_* (In_2_Se_3_)*_x_* (*x* = 0, 0.02, 0.04, 0.07). (d) Interaxial angle *α*_c_ and lattice parameter *a*_c_ obtained from Rietveld refinements based on the pseudo‐cubic lattice as a function of In_2_Se_3_ alloying content *x* [30, 33, 40, 48, 49].

Figure 3a shows the representations of the unit cell for the rhombohedral phase of GeTe. At high temperatures, GeTe crystallizes in the NaCl‐type cubic structure (space group $Fm\bar{3}m$), with Ge and Te atoms occupying two interpenetrating face‐centered cubic sublattices and thus achieving high structural symmetry [50]. Consequently, the typical XRD pattern of the cubic phase shows a single characteristic (220)_C_ diffraction peak with a *d* spacing ∼ 2.1245 Å. At room temperature, the rhombohedral phase (space group *R*3*m*) is characterized by a slight tensile distortion of the cubic phase along the body diagonal [111] direction and a decreased interaxial angle (*α*_c_) from 90° to 88.2° [51]. As a result, the originally equivalent {220} planes in the cubic phase becomes nonequivalent and the single (220)_C_ diffraction peak splits to (104)_R_ and (110)_R_ doublet reflections. Therefore, the splitting space between these two peaks can serve as a key indicator of the rhombohedral distortion degree in GeTe. The powder XRD patterns in Figure 3b,c reveal the structural evolution of (GeTe)_1‐2_ *_x_* (In_2_Se_3_)*_x_*. It can be seen that, with increasing *x*, the splitting space between the (104)_R_ and (110)_R_ diffraction peaks for *r*‐GeTe at 2*θ* ≈ 42°–44° gradually decreases, and the two peaks eventually merge into a single (220)_C_ peak when *x* = 0.07. The XRD pattern of the *x* = 0.07 sample suggests an evolution toward higher structural symmetry. However, the relatively broad diffraction peaks and the fact that the pseudocubic interaxial angle approaches 89.5° (see below), indicating that the rhombohedral distortion is substantially weakened rather than eliminated. Therefore, residual local distortion or structural inhomogeneity may persist (see Figure S5 for the TEM images of *x* = 0.07 sample), consistent with the weakened and highly diffuse phase‐transition behavior suggested by DSC.

Figure 3d shows the doping concentration dependence of the lattice parameter *a*_c_ and interaxial angle *α*_c_ under pseudocubic representations derived from the Rietveld refinement results of the XRD data. Figure S6 shows the Rietveld refinement profiles of the In_2_Se_3_‐alloyed GeTe samples. The corresponding refined lattice parameters, pseudocubic interaxial angles, *R*_wp_ and GOF are summarized in Table S1. For ease of comparison with the literature, the composition of our In_2_Se_3_ alloyed samples is normalized to the anionic lattice as Ge_(1‐2_ *_x_*_)/(1+_ *_x_*_)_In_2_ *_x_*_/(1+_ *_x_*_)_Va*_x_*_/(1+_ *_x_*_)_Te_(1‐2_ *_x_*_)/(1+_ *_x_*_)_Se_3_ *_x_*_/(1+_ *_x_*_)_, in which Va stands for the presence of Ge vacancies. The calculation methods for converting the lattice constants and interaxial angles among the pseudocubic, rhombohedral, and hexagonal crystallographic representations of *r*‐GeTe is provided in the Supporting Information (Figure S7). As shown in Figure 3d, with the increasing doping/alloying contents, the In_2_Se_3_ alloyed samples exhibit a more pronounced *a*_c_ contraction trend than those of the In‐ or Se‐doped samples, while *a*_c_ of the InSe (1:1) solid solution remains nearly unchanged. The lattice contraction can be partially attributed to the substitution of Se at the Te site, due to the smaller ionic radius of Se^2−^(1.98 Å) compared to that of Te^2−^(2.21 Å) [52]. Meanwhile, the non‐equimolar (2:3) In^3+^/Se^2−^ substitution is accompanied by the formation of Ge vacancies, which further intensifies the lattice contraction and ultimately manifests as a significant decrease in the *a*_c_. On the other hand, in contrast to In single doping, Se single doping, and In/Se 1:1 co‐doping, the In_2_Se_3_‐alloyed samples with intentionally created Ge vacancies exhibit a nearly monotonic rise in *α*_c_ as the alloying level increases similar to those of Bi/Sb and Mn doping [48, 53], revealing the indispensability of Ge vacancies in propelling the crystal sturcture of our samples to a higher symmetry.

It is well known that the rhombohedral structure of GeTe is essentially a Peierls distortion of the cubic phase along the [111] crystallographic direction, driven by the stereochemically active 4s^2^ lone‐pair electrons of Ge [54, 55, 56]. Recent studies have shown that the orientational ordering of Ge lone‐pair electrons can be weakened by introducing Ge vacancies or doping with lone‐pair‐free elements, thereby suppressing the rhombohedral distortion of GeTe and stabilizing its cubic phase [55]. Therefore, the increase in the *α*_c_ in our samples can be attributed to the combined effect of doping with lone‐pair‐free In^3+^ ions and the introduction of Ge vacancies. Meanwhile, Ge vacancies in GeTe may contribute to local lattice distortions and structural heterogeneity [57, 58]. When the long‐range coherence of these distortions is weakened, the average structure can evolve toward a more symmetric state, which may facilitate the optimization of electrical transport properties in GeTe [59].

**FIGURE 4:**
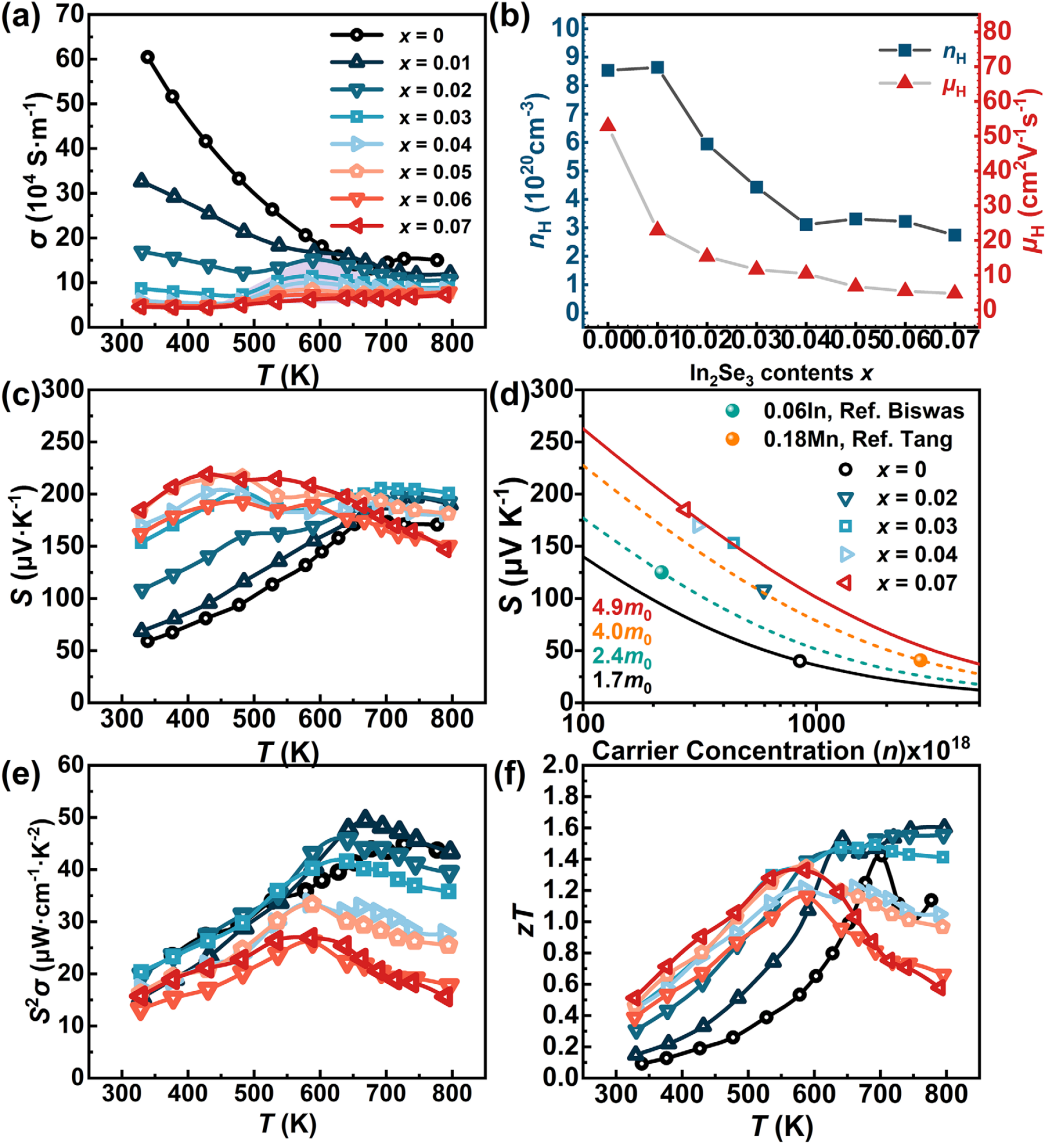
(a) Temperature‐dependent electrical conductivity (*σ*) of (GeTe)_1‐2_ *_x_* (In_2_Se_3_)*_x_*. (b) Room‐temperature Hall carrier concentration (*n*_H_) and Hall mobility (*µ*_H_) of (GeTe)_1‐2_ *_x_* (In_2_Se_3_)*_x_* samples (*x* = 0–0.07). (c) Temperature‐dependent Seebeck coefficient (*S*) of (GeTe)_1‐2_ *_x_* (In_2_Se_3_)*_x_*. (d) Carrier concentration *n*_H_‐dependent Seebeck coefficient *S* at 330 K, compared with literature results [48, 53]. (e) Temperature‐dependent power factor *S* ^2^ *σ*. (f) Temperature‐dependent thermoelectric figure of merit *zT* of (GeTe)_1‐2_ *_x_* (In_2_Se_3_)*_x_* (*x* = 0–0.07).

The electrical transport properties of (GeTe)_1‐2_ *_x_* (In_2_Se_3_)*_x_* (*x* = 0 ∼ 0.07) samples are displayed in Figure 4. Figure 4a shows the temperature‐dependent *σ* of the In_2_Se_3_‐alloyed samples. It should be noted that the room‐temperature *σ* gradually decreases with increasing In_2_Se_3_ alloying content, which can be attributed to the simultaneous decrease in carrier concentration (*n*_H_) and mobility (*µ*_H_) after In_2_Se_3_ incorporation. Under our charge‐neutral doping condition (see the defect reaction equation), the incorporation of In_2_Se_3_ should have introduced Ge vacancies without altering the hole concentration of GeTe. However, as shown in Figure 4b, when In_2_Se_3_ is initially incorporated into the GeTe matrix, the carrier concentration drops sharply, and its variation trend levels off at high doping concentrations. The initial decrease in the carrier concentration may partly originate from the redissolution of Ge precipitates facilitated by intentionally introduced Ge vacancies, and thereby reducing the hole concentration [29], while still ensuring the increasing of Ge vacancies in the system. Meanwhile, the substitution of Se for Te increases the electronegativity of the anion sublattice, which also lowers the carrier concentration of the system. The SEM images in the Supporting Information (Figure S8) further demonstrate that the amount of Ge precipitates in the GeTe matrix decreases markedly with increasing In_2_Se_3_ alloying contents. As a result, In_2_Se_3_ alloying not only introduces Ge vacancies but also successfully optimizes the carrier concentration of the system to around 3 × 10^20^ cm^−3^.

Interestingly, the *σ* of the (GeTe)_1‐2_ *_x_* (In_2_Se_3_)*_x_* samples within the medium alloying range (*x* = 0.02, 0.03 and 0.04) exhibits an anomalous hump‐like temperature dependence, which may be associated with the ferroelectric phase transition [60]. The extended temperature range of the humps in the *σ‐T* curve (indicated by the purple trapezoid in Figure 4a) is well consistent with the broadened endothermic peaks region in the DSC curves (Figure 2b), indicating that humps are also caused by the diffuse ferroelectric phase transition of the In_2_Se_3_‐alloyed samples. Furthermore, within this temperature range, the Seebeck coefficient *S* is modulated to a favorable level and remains approximately stable at ∼200 µV K^−1^ (Figure 4c). Additionally, it is worth noting that the enhanced room‐temperature *S* of our samples originate not only from the reduced carrier concentration, but more importantly from the increased effective mass. Accordingly, the Pisarenko relation between the *S* and *n*_H_ was used to evaluate the effective mass of the In_2_Se_3_ alloyed samples (Figure 4d). It is evident that In_2_Se_3_ alloying effectively increases the hole effective mass (m*) of GeTe‐based samples. The (GeTe)_0.96_(In_2_Se_3_)_0.02_ sample exhibits a m* equivalent to that of the 18% Mn‐doped sample (∼ 4.0 *m*_0_). Then, the m* further increases to 4.9 *m*_0_ with increasing alloying contents, which is much higher than that of the In single doping sample. Therefore, the increase in m* in our system cannot be solely attributed to the resonant level brought about by In doping, but also includes the effect of increased band degeneracy. Based on the above analysis, those samples achieve synergistic optimization of *σ* and *S* within the diffuse phase transition temperature range, leading to an optimized power factor (Figure 4e). Finally, as shown in Figure 4f, although the *zT*_max_ of the In_2_Se_3_ alloyed samples (only 1.60) is still not superior to that of some high‐performance GeTe‐based materials, the simultaneous optimization of electrical and thermal transport properties in a broader temperature range induced by the In_2_Se_3_ alloying enables the sample to maintain *zT*_avg_ values above 1.0 in the range of 330–625 K (Figure 2d). In fact, as a phase‐change material, the ferroelectric phase transition of GeTe may potentially affect its mechanical properties and heat cycling stability, whereas our In_2_Se_3_ solid solution material still exhibits a diffuse ferroelectric phase transition within the measured temperature range. Therefore, repeated measurements were performed on a representative sample to evaluate reproducibility. As shown in Figure S9, the repeated measurements of total thermal conductivity, power factor, and *zT* show consistent temperature‐dependent trends, with only minor deviations within the acceptable experimental uncertainty, confirming the good reproducibility and reliability of the key thermoelectric properties, which may be ascribed to the advantages of the vacancy‐induced relaxor ferroelectric phase transition over the conventional ferroelectric phase transition in terms of mechanical properties. Conventional ferroelectrics undergo a sharp first‐order phase transition that induces drastic lattice deformation and stress, leading to defects and microcracks [61]. In contrast, relaxor ferroelectrics exhibit a diffuse phase transition that proceeds gradually, resembling the progressive evolution of the dynamics of PNRs, thereby effectively avoiding stress concentration [62]. Moreover, the nanoscale multiphase coexistence and heterogeneous structure within relaxors provide an intrinsic mechanism for releasing macroscopic stress [63].

These results demonstrate that our non‐equimolar (2:3) In^3+^/Se^2−^ substitution strategy, which deliberately creates Ge vacancies under an electrical neutrality condition, can effectively broaden the ferroelectric phase‐transition temperature range as well as induce abundant polar nanoregions, thereby enabling a desirable *zT*_avg_ despite a relatively limited enhancement in *zT*_max_. However, the incorporation of the In element also deteriorates the carrier mobility, which restricts the further improvement of *zT*_max_. Therefore, future optimization should focus on retaining the advantages of Ge‐vacancy engineering while mitigating the mobility loss.

## 4 Conclusion

Relaxation in ferroelectrics arising from local lattice distortion is known to induce strong lattice anharmonicity and phonon scattering, which is highly beneficial for achieving low lattice thermal conductivity within a broad temperature range. However, this effect has historically not been extensively utilized in the field of thermoelectrics, primarily due to conflicting requirements for electrical conductivity. GeTe is a well‐established thermoelectric material, yet it suffers from excessively high hole concentrations. In this work, we demonstrate that alloying GeTe with In_2_Se_3_ introduces additional Ge vacancies. This leads to a refined hierarchical ferroelectric domain structure and a broadened temperature window for ferroelectric instability. Consequently, an ultralow lattice thermal conductivity of as low as 0.28–0.49 W m^−1^ K^−1^ has been achieved within the temperature range of 530–640 K. Furthermore, In_2_Se_3_ alloying enables optimization of both the hole concentration and band degeneracy, contributing to a marked enhancement of the power factor. Owing to the combined effects, the *zT*_avg_ of GeTe is improved to 1.02 over the temperature range of 330–625 K, highlighting the distinct advantage of harnessing ferroelectric instability in thermoelectric design.

## Author Contributions

**Yuting Zhang**: methodology, data curation, investigation, validation, formal analysis, Writing – original draft, visualization. **Yang Li**: investigation, data curation. **Jianguo Chen**: conceptualization, data curation. **Jiye Zhang**: conceptualization, methodology, data curation, investigation, validation, project administration, writing – original draft, writing – review and editing, supervision, funding acquisition, visualization. **Jun Luo**: supervision, funding acquisition, conceptualization, writing – review and editing. **Kai Guo**: methodology, data curation, validation, writing – original draft, writing – review and editing.

## Conflicts of Interest

The authors declare no conflicts of interest.

## Supporting information




**Supporting File**: advs76578‐sup‐0001‐SuppMat.docx.

## Data Availability

The data that support the findings of this study are available from the corresponding author upon reasonable request.
