# Supplementary material for: Relaxor Ferroelectricity Enables Enhanced Thermoelectric Performance in In2Se3‐Alloyed GeTe
Source: Adv Sci (Weinh). 2026 Jul 20;13(56):e76578. doi: 10.1002/advs.76578 (PMC13383686; doi:10.1002/advs.76578)
Supplement: Supplementary file 1 [file ADVS-13-e76578-s001.docx]

**Supporting Information**

**Relaxor Ferroelectricity Enables Enhanced Thermoelectric Performance in In_2_Se_3_-Alloyed GeTe**

*Yuting Zhang* ^a^, *Yang Li* ^a^, *Jianguo Chen* ^a^*, Kai Guo* ^b,c^***, Jun Luo* ^d^****, Jiye Zhang* ^a^***

^a^ School of Materials Science and Engineering, Shanghai University, 99 Shangda Road, Shanghai 200444, China

^b^ School of Physics and Materials Science, Guangzhou University, Guangzhou 510006, China

^c^ Guangdong Provincial Engineering Research Center for Materials under Extreme Service Environments, Guangzhou University, Guangzhou 510006, China

^d^ Interdisciplinary Materials Research Center, School of Materials Science and Engineering, Tongji University, Shanghai 201804, China

** Corresponding author.* E-mail: jychang@shu.edu.cn (J. Zhang)

*** Corresponding author.* E-mail: kai.guo@gzhu.edu.cn (K. Guo)

**** Corresponding author.* E-mail: junluo@tongji.edu.cn (J. Luo)

**Figure S1.** Higher-magnification TEM images and EDS elemental maps of the (GeTe)_0.96_(In_2_Se_3_)_0.02_ sample.

**Figure S2.** Wider-field EBSD and EDS characterization of the (GeTe)_0.96_(In_2_Se_3_)_0.02_ sample, including the EBSD band contrast (BC) map, inverse pole figure (IPF) maps, and EDS elemental maps of Ge, Te, In, and Se.

**Figure S3.** Sound velocities of (GeTe)_1-2_*_x_*(In_2_Se_3_)*_x_* samples at 300 K. *v*_l_, *v*_t_, and *v*_a_ denote the longitudinal, transverse, and average sound velocities, respectively.

The average sound velocity *v*_a_ was calculated from the measured longitudinal sound velocity *v*_l_ and transverse sound velocity *v*_t_ according to Equation S1:

$v_{a}=\left[ \frac{1}{3}(\frac{1}{{v_{l}}^{3}}+\frac{2}{{v_{t}}^{3}}) \right]^{-\frac{1}{3}}$	(S1)

where *v*_l_ and *v*_t_ represent the longitudinal and transverse sound velocities, respectively.

**Figure S4.** Temperature dependence of (a) total thermal conductivity *κ*_tot_, (b) electronic thermal conductivity *κ*_e_, and (c) lattice thermal conductivity *κ*_L_ = *κ*_tot_ - *κ*_e_ for (GeTe)_1-2_*_x_*(In_2_Se_3_)*_x_* samples (*x* = 0 ~ 0.07).

**Figure S5.** TEM images of the *x* = 0.07 sample at different magnifications, showing diffuse contrast and nanoscale local structural inhomogeneity.

As shown in Figure S5, the *x* = 0.07 sample still exhibits diffuse contrast and nanoscale local structural inhomogeneity. This observation suggests that residual local distortion may still exist, although the long-range rhombohedral distortion is strongly weakened. Therefore, the absence of an obvious endothermic peak in the DSC curve should not be interpreted as the complete disappearance of the ferroelectric phase transition. Instead, it more likely indicates that the transition becomes strongly weakened and highly diffuse, making the corresponding thermal signal difficult to resolve by DSC.

**Figure S6.** Rietveld refinement profiles for the room-temperature rhombohedral phase of the In_2_Se_3_-alloyed GeTe samples. The observed, calculated, and difference profiles are shown, together with the Bragg reflection positions of rhombohedral GeTe.

**Table S1.** Refined lattice parameters and reliability factors of the In_2_Se_3_-alloyed GeTe samples obtained from GSAS-II refinement.

| Sample | *a*_h_ (Å) | *c*_h_(Å) | *a*_p_ (Å) | *a*_c_(Å) | *α*_p_(º) | *α*_c_(º) | *R*_wp_(%) | GOF |
| --- | --- | --- | --- | --- | --- | --- | --- | --- |
| *x*=0.01 | 4.165 | 10.636 | 4.284 | 5.975 | 58.17 | 88.39 | 9.87 | 1.25 |
| *x*=0.02 | 4.164 | 10.597 | 4.273 | 5.966 | 58.32 | 88.52 | 10.46 | 1.43 |
| *x*=0.03 | 4.161 | 10.559 | 4.261 | 5.956 | 58.44 | 88.63 | 10.99 | 1.55 |
| *x*=0.04 | 4.154 | 10.511 | 4.246 | 5.940 | 58.57 | 88.75 | 10.00 | 1.64 |
| *x*=0.05 | 4.159 | 10.485 | 4.240 | 5.940 | 58.74 | 88.89 | 10.01 | 1.72 |
| *x*=0.06 | 4.160 | 10.444 | 4.229 | 5.933 | 58.92 | 89.05 | 13.80 | 2.23 |
| *x*=0.07 | 4.169 | 10.351 | 4.207 | 5.923 | 59.41 | 89.48 | 11.58 | 1.62 |

***Note***: The hexagonal lattice parameters are denoted as *a*_h_ and *c*_h_. The primitive rhombohedral lattice constant and interaxial angle are denoted as *a*_p_ and *α*_p_, respectively, while *a*_c_ and *α*_c_ represent the corresponding pseudocubic lattice constant and interaxial angle. The relationship between the refined hexagonal lattice parameters and the corresponding primitive rhombohedral and pseudocubic lattice parameters is further illustrated in Figure S7.”

**Figure S7.** Schematic crystal structures of GeTe at room temperature represented in the hexagonal lattice (orange), pseudocubic lattice (green), and rhombohedral lattice (pink).

To quantitatively describe the structural distortion of rhombohedral GeTe, the hexagonal lattice parameters $a_{h}$and $c_{h}$ were first converted into the rhombohedral primitive-cell parameters and then into the pseudocubic representation^1^. The relationship between the hexagonal lattice parameters and the rhombohedral lattice constant $a_{p}$and interaxial angle $\alpha_{p}$ (~ 60º) can be expressed as:

$a_{h}=2a_{p}\times\sin(\frac{\alpha_{p}}{2})$	(S2)

$c_{h}=a_{p}\times\sqrt{3(1+2\cos\alpha_{p})}$	(S3)

$a_{p}$ and $\alpha_{p}$can be determined according to the given $a_{h}$ and $c_{h}$ values by combining the above two equations and solving numerically. Furthermore, the pseudocubic lattice constant $a_{c}$ and related interaxial angle $\alpha_{c}$ (~ 90º) can be calculated using the following relationships:

$a_{c}=\sqrt{a_{p}^{2}+a_{h}^{2}}$	(S4)

$\alpha_{c}=2\arcsin(\frac{a_{h}}{a_{c}})$	(S5)

Accordingly, $\alpha_{c}$ can be directly calculated using the following equation:

$\alpha_{c}=2\arcsin[\frac{2}{\sqrt{4+\csc[\frac{\alpha_{p}}{2}]^{2}}}]$	(S6)

**Figure S8.** SEM morphology and EDS elemental maps for *x* = 0, 0.02, 0.06 samples.

**Figure S9.** Repeatability of the thermoelectric properties for the representative (GeTe)_0.92_(In_2_Se_3_)_0.04_ sample, including the power factor *S*^2^*σ*., total thermal conductivity *κ*_tot_, and *zT* measured over repeated runs.

**SPB model:**

The SPB model equations used to calculate the Hall carrier concentration (*n*_H_), Seebeck coefficient (*S*), Hall factor (*r*_H_), and density-of-states effective mass (*m*_d_*) are expressed as follows:

$n_{H}=\frac{1}{eR_{H}}=\frac{8\pi(2m_{d}^{*}k_{B}T)^{3/2}}{3h^{3}}\frac{(r+3/2)^{2}}{(2r+3/2)}\frac{F_{(r+1/2)}^{2}(\eta)}{F_{(2r+1/2)}(\eta)}$	(S7)

$r_{H}=\frac{3}{2}\frac{(2r+3/2)}{(r+3/2)^{2}}\frac{F_{1/2}(\eta)F_{(2r+1/2)}(\eta)}{F_{(r+1/2)}^{2}(\eta)}$	(S8)

$F_{i}(\eta)=\int_{0}^{\infty} \frac{x^{i}}{1+e^{x-\eta}}dx$	(S9)

$S=\frac{k_{B}}{e}[\frac{(r+5/2)F_{(r+3/2)}(\eta)}{(r+3/2)F_{(r+1/2)}(\eta)}-\eta]$	(S10)

$m_{d}^{*}=\frac{h^{2}}{2k_{B}T}[\frac{n_{H}r_{H}}{4\pi F_{1/2}(\eta)}]^{2/3}=N_{v}^{2/3}m_{b}^{*}$	(S11)

where *k*_B_ is the Boltzmann constant, *h* is the Planck constant, *e* is the elementary charge, *n*_H_ is the Hall carrier concentration, *R*_H_ is the Hall coefficient, *r*_H_ is the Hall factor, *η* is the reduced Fermi level, *F_i_*(*η*) is the Fermi integral of order *i*, *N*_v_ is the band degeneracy, *m*_d_^*^ is the electronic density-of-states effective mass, *m*_b_^*^is the band effective mass, *r* is the scattering parameter, and *T* is the absolute temperature.

(1) T. Chatterji; C. M. N. Kumar; U. D. Wdowik. "Anomalous temperature-induced volume contraction in GeTe," *Physical Review B 91* (2015): 054110, <http://doi.org/10.1103/PhysRevB.91.054110>.
